# Supplementary material for: High prevalence of intestinal parasite infestations among stunted and control children aged 2 to 5 years old in two neighborhoods of Antananarivo, Madagascar
Source: PLoS Negl Trop Dis. 2021 Apr 20;15(4):e0009333. doi: 10.1371/journal.pntd.0009333 (PMC8087024; doi:10.1371/journal.pntd.0009333)
Supplement: Attachment — Submitted filename: Response_to_reviewers.docx [file pntd.0009333.s002.docx]

Madagascar, November 27^th^, 2020

Arunasalam Pathmeswaran

Associate Editor

PLOS Neglected Tropical Diseases Journal

Marco Coral-Almeida

Deputy Editor

PLOS Neglected Tropical Diseases Journal

**Manuscript Number**: PNTD-D-20-01478

Dear Editors,

Thank you for reviewing our submission entitled “High prevalence of intestinal parasite infestations among stunted and control children aged 2 to 5 years old in two neighborhoods of Antananarivo, Madagascar” (PNTD-D-20-01478). We are grateful both to the editorial team and to the reviewers for their thoughtful feedback. In response to these comments, we have substantially revised the manuscript to address statistical and technical concerns. We feel these revisions strengthen the presented analysis and hope you will consider this revised manuscript for publication in PLOS NTD Journal.

The detailed revisions are highlighted in the point-to-point response below. We thank you again for your time and attention.

Sincerely,

HABIB Azimdine

**Response to Reviewers – Manuscript PNTD-D-20-01478**

Reviewer Comments

Reviewer #2: Comments to the authors

In this paper Habib et al. attempt to describe the occurrence of enteric parasites, including helminthic and protist species, and their association with severe and moderate stunting and other risk factors in children under five years of age living in deprived areas of Antananarivo, Madagascar. Surveyed paediatric populations included community children and children seeking medical attention at hospital settings, and were correctly matched by sex, age, neighbourhood and sampling season. Enteric pathogens were detected by conventional microscopy (MIF and KK) methods and qPCR. Near 100% of the surveyed children were infected by at least one parasitic species. No differences were found between stunted and non-stunted children according to their infection status by enteric parasites. No molecular studies were conducted to ascertain the molecular diversity (in terms of genotypes and sub-genotypes) of the main protist species found, particularly G. intestinalis, Cryptosporidium spp., and E. bieneusi. The study is relevant because provides important epidemiological data regarding the presence of enteric parasites of public health relevance. However, there are a number of issues that need attention and clarification and that probably have biased the results obtained, their interpretation, and the conclusions reached by the Authors.

**Mayor issues**

Please find the remarks below:

1-Introduction section, line 74-75: please mention here *Cryptosporidium* spp. Please note that Cryptosporidium is second only to rotavirus in causing diarrhoea and death in children younger than five years in developing countries, particularly in Sub-Saharan Africa. See for instance Kotloff et al. Lancet. 2013;382:209-22 or Sow et al. PLoS Negl Trop Dis. 2016;10:e0004729.

Thank you for the comment. The lines 74-75 in the original paper become lines 79-80 in the modified one and we added: primarily *Cryptosporidium* spp. but also to a lesser extent *Giardia intestinalis* and *Entamoeba histolytica* are also of considerable public health importance [5-8].

2-Introduction section, lines 76-77: this statement may be misleading and should be rephrased. Please note that large case-control epidemiological studies conducted in Africa have demonstrated that *G. intestinalis* was more common in controls than in cases. See for instance Becker et al. Clin Microbiol Infect. 2015;21:591.e1-10; Breurec et al. PLoS Negl Trop Dis. 2016;10:e0004283; Tellevik et al. PLoS Negl Trop Dis. 2015;9:e0004125; and Kotloff et al. Lancet. 2013;382:209-22.

The lines 76-77 in the original paper become lines 80-82 in the modified one. We have rephrased like this: For instance, among the main infectious diarrheagenic parasites, *Cryptosporidium* spp. results in the most deaths among children < 5 years of age [7].

3-Introduction section, lines 81-82: please note that these very same effects have also been described in children infected with *G. intestinalis, Cryptosporidium* spp., and *E. histolytica*. See for instance Berkman et al. Lancet. 2002;59:564-571; Carvalho-Costa et al. Rev Inst Med Trop São Paulo. 2007;49:147-153; and Mondal et al. Trans R Soc Trop Med Hyg. 2006;100:1032-1038.

The lines 81-82 in the original paper become lines 106-107 in the modified one of the introduction section and we have removed this statement “particularly STHs”.

4-Introduction section, lines 106-108: please note that *E. histolytica* cysts are morphologically indistinguishable from other non-pathogenic *Entamoeba* species including *E. dispar, E. moskowki*, and *E. Bangladeshi*. Unambiguous detection of *E. histolytica* should be based on species-specific methods such as PCR. Please notice that incorrect diagnosis of *E. histolytica* by microscopy examination has led to estimating wrong prevalence rates for this pathogen. See for instance Efunshile et al. Am J Trop Med Hyg. 2015;93:257-62. Amend. Same comment for lines 281-282, 324-325, and 328. Please provide the prevalence rate of *E. histolytica* taking qPCR (not microscopy) as diagnostic method. This should must be conducted through the whole manuscript including the statistical analyses in Table 6, Figure 3, and Figure 4.

In lines 106-108 of the introduction section, as well as lines 281, 282, 324, 325 and 328 of the results section, and in the whole manuscript, texts, results figures, table and part of discussions were amended as recommended. These changes led to new results about *E. histolytica*. Indeed, when taking into account the prevalence rate of *E. histolytica* by Real-time PCR, we have found an association between *E. histolytica* and moderately stunted children and also between untreated water. See details below.

Lines 106-108 in the original paper becomes line 131-135 in the modified one: For *Entamoeba histolytica* suboptimal sensitivity of the microscopy-based techniques which is about 60%, [29] and the inability to distinguish potentially pathogenic *Entamoeba histolytica* from morphologically identical but nonpathogenic *Entamoeba dispar*, *Entamoeba moshkovskii*, and other quadrinucleate cysts of *Entamoeba* has been demonstrated [29].

Lines 281, 282 in the original paper in the text were amended and results can be found in Table 2. The text now reads as follows in lines 316-318 in the modified paper: Regarding nutritional status and infestation, all children were equally likely to show a parasitic infestation except *E. histolytica*, which found significantly associated between stunted and nonstunted children (p=0.02) Table 2.

Lines 324-325 in the original paper becomes line 349 and changed was made in the Fig 3.

Line 328 in the original manuscript that describes comparison between microscopy result and real-time PCR for *E. histolytica* was amended and line becomes 351 in the modified paper.

Lines 371-438 in the result section in the original paper become lines 337-345 in the modified one and now read as follows: “Multivariate logistic regression analysis showed that drinking untreated water was a risk factor for *Giardia intestinalis* (*G.intestinalis*) (AOR = 1.85; 95% CI, 1.1–3.09, p=0.019) and *Entamoeba histolytica* (*E.histolytica*) (AOR=1.9; 95% CI, 1.07-3.38, p=0.028) infestations. Also, multivariate logistic regression analysis showed association between *E. histolytica* and moderately stunted children (AOR= 0.37; 95% CI, 0.2-0.71, p=0.002). In addition, living on an uncemented soil (AOR = 1.85; 95% CI, 1.18–2.09, p=0.008) was associated with *Trichuris trichiura* (*T.trichiura*) infestation as was being older (children from 4 to 5 years old (AOR = 3.2; 95% CI, 2.04–5.01, p=0.001) compared to children aged 2 to 3 years old) (Table 5).

5-Introduction section: please provide a paragraph summarizing the current epidemiological scenario of IPIs in Madagascar. Provide data on the range of reported prevalences, populations (general, paediatric, clinical, etc.) surveyed, geographical areas investigated and any other information (e.g. seasonality) that may be relevant to understand the distribution and transmission of these infections.

A paragraph summarizing epidemiological scenario of IPIs in Madagascar was added and take lines 85-103:

In different regions of Madagascar a number of research studies have been conducted on the prevalence of IPI among children using microscopy-based techniques for the identification of IPI. A cross-sectional study done in remote villages within the Ifanadiana district of Madagascar in 2016 revealed an overall IPI prevalence of 92.5% for intestinal parasites. Among children between 5 and 14 years old, four different types of IPs were detected and *Trichuris trichiura* was the most commonly encountered parasite (84.6%) followed by *Ascaris lumbricoides* (72.4%) [14]. Three different types of intestinal parasites (IPs) were identified in 14 districts of Madagascar in 2008-2009 from children under five years of age suffering or not of diarrheal disease. The overall prevalence was 36.5% and *Giardia intestinalis* was the dominant IP (12.6%) followed by *Trichomonas intestinalis* (6.2%) and *Entamoeba histolytica* (2.0%) [15]. A study in rural areas of Moramanga and Morondava, Madagascar in 2014-2015 indicated 23.6% overall prevalence and the most common parasites identified in Moramanga was *Ascaris lumbricoides* (16.1%), followed by *Trichuris trichiura* (3.8%). In Morondava, *Entamoeba coli* (6%) and *Hymenolepis nana* (4%) were the most prevalent parasites [16].

Epidemiological information regarding the prevalence and associated factors of IPIs and other diarrheal causing pathogens among children less than 5 years of age is not available in the other regions of the country including Antananarivo, which was our study area

6-Line 124: what is the Afribiota study? Is this survey taking advantage of that other survey, or is it part of it? What were the main goals of the Afribiota study? Information provided in lines 130 and below is insufficient. Please clarify.

More detail was added about the Afribiota study in lines 151-156: AFRIBIOTA is a case-control study on stunted children in Antananarivo, Madagascar and in Bangui, Central African Republic carried out from December 13th, 2016 to March 20th, 2018 [32]. The main objective of AFRIBIOTA was to describe the intestinal dysbiosis observed in the context of stunting and to link it to pediatric environmental enteropathy (PEE), a chronic inflammation of the small intestine.

And in lines 161-163:

in each country, 460 children aged 2–5 years with no overt signs of gastrointestinal disease were recruited (260 with no growth delay, 100 moderately stunted and 100 severely stunted) [32].

7-Line 188: please clarify whether the PCR-detection of *Cryptosporidium* species was specific for *C. parvum* or included also other species such as *C. hominis* and *C. meleagridis.* This is important as *C. hominis* is the *Cryptosporidium* species most commonly identified in humans globally.

In line 188, yes it’s only *Cryptosporidium parvum* that was targeted. Line in modified paper becomes 221-223. We have given a table (Table 1) that summarize primers and probe uses in this study.

To better reflect this fact, the text was changed as follows: We used parasite-specific primers and probes from published studies with some modifications to fluorochromes. The respective primers and their references are listed in Table 1.

8-Line 284: please replace “*Blastocystis hominis*” by “*Blastocystis* sp.” as this heterokont protist is not human-specific. See Stensvold et al. Trend Parasitol. 3007;23:93-96. Please amend here and through the whole manuscript.

Line 284 in the original paper becomes 306 in the modified one and we replaced *Blastocystis hominis* as suggested by *Blastocystis* sp. and the change appear in the table 2 and we change also in the whole manuscript.

9-Results section, lines 269-286: please replace these paragraphs with a new Table showing the diversity and frequency of IPIs in the two neighbourhoods surveyed. Information provided in the main body of the text should only refer to the most important data of the Table.

We modified lines 269-286 in the original paper and these lines become lines 300-307 in the modified one in the text and added the suggested table. The text now reads as follows: Children were equally infested by intestinal protozoa (88.5%, n = 363) and helminths (88.3%, n = 362). Overall, 16 different species of IPIs were detected. The predominant IPIs were *G. intestinalis* (79.5%, n = 326), *A. lumbricoides* (68.3%, n=280) and *Trichuris trichiura* (*T. trichiura*) (68.0%, n=279). *E. bieneusi* was found in 136 children (33.1%) and constituted the predominant microsporidia species. This species were found significantly more often in Andranomanalina Isotry compared to Ankasina (p=0.02). Table 2 shows the prevalence of all detected IPIs, their frequencies in stunted and nonstunted children and their frequencies in each of the two community settings.

10- Results section, lines 287-291: same comment as above for the coinfections detected

Lines 287-291 of the results section in the original paper become 312-317 in the modified one and now read as follows: Polyparasitism was more common (91.5%, n=375) than monoparasitism (4.9%, n=20). Infestations with the three parasites *G. intestinalis, T. trichiura* and *A. lumbricoides* was diagnosed in 40 severely stunted (9.8%), 38 moderately stunted (9.3%) and in 96 nonstunted children (23.4%). Table 3 summarizes the occurrence of polyparasitism in stunted and nonstunted children. All children, regardless of their nutritional status, were equally likely to display polyparasitism.

11-Results section. No attempts were conducted to genotype samples with a positive result to *G. intestinalis, Cryptosporidium* spp. and *E. bieneusi*. Although this was not the primary goal of the survey, unravelling the genetic diversity of these pathogens is a task that should be conducted in future studies. This should be stated as a limitation of the study in the Discussion section in lines 482-488.

Indeed no genotyping was conducted in this study. This limitation was added in the discussion section in lines 531-534 in the modified paper as follows: Furthermore, in this study, no attempts were conducted to genotype samples with a positive result to *G. intestinalis*, *C.* *parvum* and *E. bieneusi*. Although this was not the primary goal of the survey, unravelling the genetic diversity of these pathogens is a task that should be conducted in future studies.

12-Discussion section: please provide more information about previous epidemiological studies conducted in Madagascar, e.g. in lines 378. Please make every effort to put obtained results in the right context by comparing them with previous data in the country. If possible, compare prevalence rates, detection methods, population types, and geographical areas.

More information was added in the discussion section about previous epidemiological studies conducted in Madagascar and this will be found in lines 397-411. The paragraph now reads as follows: However, the prevalence of protozoa, such as *G. intestinalis* (79.5%), was considerably higher than that found in other studies [43, 44] conducted in Madagascar, where the authors found prevalence of 24.4% and 16.4% respectively. The majority of the studies conducted in Madagascar was carried out in the western region and prevalence of *G. intestinalis* varied between villages. For example, an epidemiological study of the etiology of infant diarrheal disease conducted in 2008-2009 in 14 districts in Madagascar showed 26.2%, 20.2% and 18.1% in Maevatanana, Morondava and Majunga, respectively in the western region [41].

The difference in prevalence compared to our study may be due to the methods used rather than the study area: previous studies performed in Madagascar only used microscopy mainly the MIF technique to detect *G. intestinalis* while we used both, microscopy and real-time PCR, which has a much higher sensitivity. This is exemplified through the fact that the prevalence of *G. intestinalis* in our study was 21% when using the MIF technique, which is almost identical to the prevalences, reported in other regions of the country while it was 78% by the real-time PCR (Fig 3).

13-Discussion section, line 445: for waterborne transmission of *G. intestinalis* and other protist parasites see Muadica et al. Clin Microbiol Infect. 2020. doi: 10.1016/j.cmi.2020.05.031. This paper should be mentioned and adequately discussed here.

Lines 445 in the original paper become lines 480-488 in the modified one and we have amended the text as follows: The present finding showed a significant association between *G. intestinalis* prevalence and untreated water (AOR = 1.85, 95% C. I = 1.1–3.09, p = 0.019), which is similar to the results obtained by Osten et al. [55] and Muadica et al. [56]. In a study carried out in Argentina, it was determined that intestinal parasite frequencies detected in various sociocultural areas were related to contaminated water resources by the parasites, as well as insufficient health conditions [57]. The study of Muadica et al. showed that drinking river/stream as a primary or secondary source of water was identified as a risk association for *G. intestinalis* and water chlorination/ boiling reduced the odds of this species in children.

And we have added in lines 480-486 a text as follow: Moreover, *E. histolytica,* a waterborne transmission like *G. intestinalis* was statistically significantly associated with untreated water (AOR=1.9; 95% CI, 1.07-3.38) (Table 5) in this study. This shows the importance of source and quality of water as factor of transmission of infectious pathogens. Indeed, during the present study, sewage and toilet wastewater were often found freely flowing to the water sources (e.g., wells), which is a concern for possible waterborne transmission like *G. intestinalis* and *E. histolytica* infestation [54].

14-Discussion section, lines 461-464: please add to this list the potential presence of PCR inhibitors in the extracted and purified genomic DNAs.

In lines 461-462 in the original paper that becomes line 505-507 in the modified one, a statement about potential PCR inhibitor was added: The presence of microscopy-positive, PCR negative samples could also be due to potential presence of PCR inhibitors in the extracted and purified genomic DNA

**Minor issues**

1-Line 32: *Giardia intestinalis* should be italicized.

This was corrected

2-Line 33: *Entamoeba histolytica* should be italycised.

This was corrected

3-Line 34: *Ascaris lumbricoides* should be italicised.

This was corrected

4-Line 58: please remove “or more” (rephrase as “…with at least one parasite species”).

Line 51 in the original paper becomes line 61 in the modified on and this was removed

5-Lines 156 and 157: what was the difference between the variables “household waste” and “waste”? Please clarify. Also, what was the meaning of the variable “cooked”? Please clarify.

Line 156-157 in the original paper becomes 188-189 and Household waste is in fact “sewage”. We changed the wording throughout the text.

Waste is “garbage”. We changed the wording throughout the text

Cooked was amended for “location of the kitchen area (inside house or outside house)”

6-Line 160: mothers (lower case).

Line 160 becomes line 192 and this was corrected

7-Line 175: “triturated” is not probably the best term to be used here. Amend.

Line 175 in the original paper becomes line 206 in the modified one and triturated was amend on “mixed”

8-Line 221: *Escherichia coli* in full and italicised. Species names should be named in full the first time they are mentioned in the text.

Line 221 in the original paper becomes line 251 and this was corrected

9-Lines 275 and 435: *Giardia intestinalis*. In full at the beginning of a new sentence.

This was corrected. Line 435 in the original paper becomes 469 in the modified one

10-Line 494: E. spp.? Please indicate the genus.

Line 494 in the original paper becomes 540 and *E*. spp means *Encephalitozoon* spp. and this was corrected

Reviewer #3:

This manuscript arising from AFRIBIOTA study presents that prevalence of intestinal parasites are higher among stunted and control children and compares the detection rate of various methods.

Thank you for the comments

Figure Files:

While revising your submission, please upload your figure files to the Preflight Analysis and Conversion Engine (PACE) digital diagnostic tool, <https://pacev2.apexcovantage.com>. PACE helps ensure that figures meet PLOS requirements.

Figures were submitted in PACE tool and have been converted to the format required by PLOS journal.

Modifications have been made on line 5 and 6 by adding: ^#b^, ^#c^

Addresses were added in line 17-18 as follow:

^#b^ Current address: Human and Animal Health Unit, Swiss Tropical and Public Health Institute, Basel, Switzerland & University of Basel, Switzerland,

#c Current address: Center for Microbes, Development and Health (CMDH), Institut Pasteur of Shanghai, China
