# Supplementary material for: High prevalence of intestinal parasite infestations among stunted and control children aged 2 to 5 years old in two neighborhoods of Antananarivo, Madagascar
Source: PLoS Negl Trop Dis. 2021 Apr 20;15(4):e0009333. doi: 10.1371/journal.pntd.0009333 (PMC8087024; doi:10.1371/journal.pntd.0009333)
Supplement: Attachment — Submitted filename: Reponse_to_reviewers.docx [file pntd.0009333.s003.docx]

Madagascar, February 18^th^ 2021

Arunasalam Pathmeswaran

Associate Editor

PLOS Neglected Tropical Diseases Journal

Marco Coral-Almeida

Deputy Editor

PLOS Neglected Tropical Diseases Journal

**Manuscript Number**: PNTD-D-20-01478R1

Dear Editors,

Thank you for your second round of revision of our submission entitled “High prevalence of intestinal parasite infestations among stunted and control children aged 2 to 5 years old in two neighborhoods of Antananarivo, Madagascar” (PNTD-D-20-01478R1). We are grateful both to the editorial team and to the reviewers for their thoughtful feedback. In response to these comments, we have substantially revised the manuscript. We hope you can now accept this revised manuscript for publication in PLOS NTD Journal.

The detailed revisions are highlighted in the point-to-point response below. We thank you again for your time and attention.

Sincerely,

HABIB Azimdine

**Response to Reviewers – Manuscript PNTD-D-20-01478R1**

Reviewer 1

1-Lines 356-357 (Figure 3): Given that infections’ prevalence are calculated as the ratio between the number of cases and the number of individuals in the population, I am not clear about how the error bars in Figure 3 were calculated. Please clarify.

Thank you for the comment. Error bars were calculated automatically using Excel software. However to avoid confusion, we have preferred to remove the error bars in the Figure 3.

2-Please revise the writing of the parasite species throughout the text. In particular, use full binomial name only the first time a given species is mentioned. After that, abbreviate the genus as the initial followed by a full stop. Since the latter form is not actually an abbreviation, but a consensus to use species’ scientific names in a text, it does not need to be specified in brackets, as in lines 218-221. Moreover, please do not abbreviate Encephalitozoon spp. as E. spp.

We have revised all the name of species in the paper and all were rewritten according the suggestion of the reviewer.

3- Line 222: Please indicate the probe format (e.g., TaqMan).

The line 222 become lines 220 in the modified manuscript. The probe format was added (TaqMan).

4- Line 232: Please provide centrifugation speed in x g.

The line 232 becomes lines 231 in the modified manuscipt and we have provided centrifugation speed in g measure as recommended.

5- Lines 280-281: The abbreviations for severely, moderately and non-stunted should have been introduced in lines 163 and 166, where these words are used for the first time. From there to the end of the text use the abbreviature (e.g., in line 287). The same comment applies to other abbreviations such as EPG (line 383), IPIs (line 395) or STH (line 402), all of which had been already used before.

Lines 280-281 become line 278 in the modified manuscript and we used abbreviations here as suggested.

Line 383 becomes line 380 in the modified manuscript and EPG was used rather than the full name as recommended.

Line 395 becomes line 391 in the modified manuscript and IPIs was used rather than the full name as recommended.

Line 402 becomes line 398 in the modified manuscript and STH was used rather than the full name as recommended.

6- Line 293: In order to maintain the text consistency, please add the p-value for differences between female and male participants. Same in lines 325 (for age group) and 328 (for mother’s educational level).

Line 293 becomes line 290 in the modified manuscript and the p-value between female and male was added as recommended.

Line 325 becomes line 321 in the modified manuscript and the p-value between age group was added as recommended.

Line 328 becomes line 324 in the modified manuscript and the p-value for mother’s educational level was added as recommended.

7- Line 325: Indicate the meaning of AOR and CI the first time that these abbreviations are used in the text. Moreover, keep the same format throughout the text, i.e., indicate CI always in, or outside, brackets.

Line 325 becomes line 321 in the modified manuscript and the write out of the abbreviations of AOR and CI were added. Moreover, we have chosen to put the confidence intervals always in the brackets as suggested and checked for consistency throughout the text.

8- Line 340: In order to maintain consistency with the header of the previous subsection, here it should be “Potential risk […]”.

Line 340 becomes 337 in the modified manuscript and “Potential” is added in the subsection title as suggested

9- Line 365: Add “target” before “DNA loads”.

Line 365 becomes line 361 in the modified manuscript and the word “target” was added as recommended

10- Line 354: Please replace “between” by “determined by”

Line 354 becomes 351 in the modified manuscript and the word “between” was replaced by “determined by” as recommended

11- Lines 373-378: Please re-write caption for Fig 4 to explain what is shown in the figure, but without describing the results. Please indicate the results of which species are shown in each panel (A, B and C) and clarify that what is compared in each graph are the Ct values obtained in PCR positive samples for microscopy positive and negative samples.

Line 373-378 become line 369-373 in the modified manuscript and caption for Fig 4 was re-written as follow: “**Microscopy versus real-time PCR positive results for *G. intestinalis*, *A. lumbricoides* and *E. histolytica***. Boxplot for A), *G. intestinalis*, for B) *E. histolytica* and for C) *A. lumbricoides*. X axis of all boxplot indicated microscopy result of which sign “-“ means negative result and sign “+” positive result and Y axis indicate Ct value of the real time PCR”.

12- Figure 4C: Keep the same order and colour used for positive and negative samples in panels A and B.

This was corrected

13- Line 382: Rather than a positive correlation, Figure 5 shows an inverse relationship between the number of *A. lumbricoides* EPG of faeces and the Ct values determined by qPCR. This is indeed the expected result, because based on the principles of the qPCR, the higher the parasite burden (i.e., target DNA amount) is, the lower the Ct value should be. Revise this also in line 537.

This was revised and the word “positive” was removed from the text and the sentence adapted as follow: Although many *A. lumbricoides* infections are missed by KK (Fig 5), there was a correlation between the EPG count measured by KK and the Ct values measured by real-time PCR (r = 0.13, p=0.013)

line 537 becomes line 528 in the modified manuscript and the word “positively” was replaced by “inversely” and the sentence now reads as follows: “We have shown in Fig. 5 that KK and real-time PCR measures are inversely correlated for *A. lumbricoides* (r = 0.13, p=0.0131), suggesting that DNA concentrations calculated against a standard curve for real-time PCR can be used as a quantitative measure of infection intensity.”

14- Figure 5: It is curious to me that all KK samples positive for A. lumbricoides showed mean EPG of faeces equal to either 1,000 or 10,000. How could this be explained?

This is a conversion of the intensity of *A. lumbricoides* following the WHO guide in order to correctly present the data in the database.

Indeed, 100 EPG in the figure means 1-4999 EPG observed on KK and thus low intensity. 1000 EPG in the figure means 5000-49999 EPG observed on KK and therefore moderate intensity and 10000 EPG in the figure, means more than 50000 EPG observed on KK and therefore high intensity. Thus, the values 2000, 4000, 6000 and 8000 are scales provided by the R software. Consequently, the intensity presented in the figure is expressed by 100, 1000 and 10000 EPG which means low, moderate and high intensity for *A. lumbricoides*.

15- Line 385: The adjective “major” is confusing here; please rephrase.

Line 385 becomes line 379 in the modified manuscript and the term “major” was replaced by “the majority by”

16- Line 419: “were” instead of “was”.

Line 419 becomes line 411 in the modified manuscript and the sentence now reads as follows: The majority of the studies conducted in Madagascar were carried out in the western region and prevalence of *G. intestinalis* varied between villages.

17- Line 431: “disease” instead of “infection”.

Line 431 becomes line 424 in the modified manuscript and the sentence now reads as follows: Although *E. coli* and other nonpathogenic parasites detected in this study do not cause disease, their presence indicates fecal-oral, mostly waterborne transmission in the host, which is an indicator for the general assessment of the hygiene status of the area [46].

18- Line 433 (and through the discussion): Provide references. Same in lines: 444, 467, 508, 523, 525, 528, 543.

Line 433 becomes 426 in the modified manuscript and line 444 becomes 437, line 467 becomes 459, line 508 becomes 500, line 523 becomes 514, line 525 becomes 516, line 528 becomes 520 and line 543 becomes 535. All references were added as recommended.

19- Line 401 (and through the discussion): Do not repeat numeric results, neither reference to figures, in the discussion. Same in lines: 403, 439-40, 464-5, 475, 483, 485, 489, 500, 519, 530, 536-7, 565-6.

Line 401 becomes line 395 in the modified manuscript and line 403 becomes line 396, line 439-440 become 432, line 464-465 become line 457, line 475 becomes line 466, line 483 becomes line 476, line 485 becomes line 476, line 489 becomes line 481, line 500 becomes line 491, line 519 becomes line 509, line 530 becomes line 521, line 536-537 become line 527 and line 565-566 become line 556-557 and all numeric results and reference to figures were removed as suggested. Also, numeric results and figures references were removed through the discussion.

20- Line 487: Please replace “isolated” by “identified”.

Line 487 becomes line 479 in the modified manuscript and this was corrected.

21- Line 490: Please indicate where the studies by Osten and Muadica were conducted.

Line 490 becomes lines 481-482 in the modified manuscript and areas that these studies were conducted were added. The sentence now reads as follows: “The present finding showed a significant association between *G. intestinalis* prevalence and untreated water which is similar to the results obtained by Osten et al. in Manisa, Turkey [59] and Muadica et al. in central Mozambique [60]”.

22- Lines 498-500: A word seems to be missing in this phrase.

Lines 498-500 become line 490-491 and the words “species such as” were added. The sentence now reads as follows: “Moreover, *E. histolytica*, a waterborne species such as *G. intestinalis*, was statistically significantly associated with untreated water (Table 5) in this study.”

23- Lines 503-504: A word seems to be missing in this phrase.

Lines 503-504 become line 494-495 and the word “parasites” was added. The sentence now reads as follows: “Indeed, during the present study, sewage and toilet wastewater were often found freely flowing to the water sources (e.g., wells), which is a concern for possible waterborne parasites transmission like *G. intestinalis* and *E. histolytica* infestation.”

24- Line 506: “STH” instead of “SHT”.

Line 506 becomes line 498 in the modified manuscript and this was corrected.

25- Lines 512-516: “in this study” is repeated twice in the same sentence.

Lines 512-516 become lines 504-507 and the second “this study” was replaced by “we”. The sentence now reads as follows: “In this study, in addition to investigating the prevalence infection levels of intestinal parasites among stunted and nonstunted children, we also aimed to compare the diagnostic accuracy of microscopic techniques to determine *A. lumbricoides*, *G. intestinalis* and *E. histolytica* and the diagnostic accuracy by real-time PCR”

26- Line 572: Non-pathogenic parasites could also be diagnosed by PCR if desired; therefore, this does not add a value to the use of microscopy-based techniques.

We have removed the paragraph from the text.

27-Please revise tables and figures to make sure full names of parasites are provided the first time they are mentioned in a given table/figure, and that they are always written using italics. Similarly, double check that all abbreviations used in tables/figures are properly defined in the table headers or footnotes, or in the figure legends.

All were revised and corrected as recommended

Minor comments

Reviewer #2:

1-Line 98: please note that E. coli is considered a commensal rather than a parasitic species. Please amend.

Line 98 becomes line 97 and this was amended as follow: word “commensal” was added and the sentence is now reading: “In Morondava, the commensal *Entamoeba coli* (6%) and *Hymenolepis nana* (4%) were the most prevalent parasites.”

2-Line 129: “…fecal smears after fecal concentration.”

Line 129 becomes line 128 in the modified manuscript and this was corrected.

3-Line 131: *E.histolytica*. Once the species has been fully named the first time it appears in the text, please use the abbreviated form. Same comment for other parasite species that are mentioned through the whole manuscript.

This was corrected throughout the text

4-Line 138: [26,30,31]? Please double check PLoS NTD editing style for references

Line 138 becomes line 137 in the modified manuscript and this was corrected

5- Line 167: Standard Deviation (SD).

Line 167 becomes line 166 and this was corrected

6- Line 409: =<3%? Do you mean ≤3%? Please clarify

Line 409 becomes line 402 and this mean ≤ and that was corrected.

Figure Files:

While revising your submission, please upload your figure files to the Preflight Analysis and Conversion Engine (PACE) digital diagnostic tool, <https://pacev2.apexcovantage.com>. PACE helps ensure that figures meet PLOS requirements.

Figures were submitted in PACE tool and have been converted to the format required by PLOS journal.

We have added two persons on the list of AFRIBIOTA Investigators because of their contribution to the project: Milena Hasan and Darragh Duffy.
